# Meetings of Societies

**Published:** 1894-03

**Authors:** 


### MEETINGS OF SOCIETIES.

### Bristol Medico-Chirurgical Society.

*December* 13*th*, 1893.

Mr. J. Greig Smith, President, in the Chair.

The evening was devoted to a discussion on Intussusception, introduced by a paper by Mr. Arthur W. Prichard. (See page 1.)

Mr. Paul Bush was able to call to mind nine cases of intussusception occurring during the last few years in patients at the Bristol Royal Infirmary. Seven of these were treated without operation, and nearly all of them were given one or more rectal injections or inflations: death resulted in all seven. Of the two cases operated upon, the first was a child four months old, with symptoms of intussusception which had commenced at least three days before. Injections were tried and failed; abdominal section was performed. An intussusception of ileum into ileum was found to be so adherent that it could not be reduced. The whole mass was removed, and the two divided ends of the intestinal canal were fixed to the abdominal incision, with the idea of forming an artificial anus. Death resulted within twenty-four hours after operation from peritonitis. It is probable that if this case had been operated upon earlier, when the adhesions were less firm, the gut might have been reduced. The second case operated on was a patient just under four months old, who was admitted under Mr. Bush's care on May 20th, 1890, with the usual symptoms and physical signs of intussusception. Vomiting and passage of bloody mucus had commenced about eight hours previously. Large injections of warm water by means of Lund's apparatus, with the patient inverted, together with manipulations of the abdomen, were tried and repeated several times during the day of admission, without any relief of symptoms. On the 21st the child was given chloroform, and abdominal section in the middle line was performed. With a little manipulation a mass of intestine from the left side of the abdomen was brought out through the wound, when some small intestine was clearly seen passing into descending colon, and the vermiform appendix, cæcum, ascending and transverse colon were out of sight. By means of very gentle traction on the small intestine, but chiefly by pressure on the distal end of the swelling, the intestine was slowly "shelled" out from below upwards, when it was found that about five inches of the termination of the ileum, with the ileo-cæcal valve and vermiform appendix (which was long), together with the ascending and transverse colon, were invaginated into the descending colon and rectum. The colon was found to be only very loosely fixed to the back of the abdominal cavity, which allowed for this extensive invagination. There was some slight general peritonitis, but the adhesions of the invaginated gut were only of very soft lymph. On restoration of the continuity of the gut, an irregular depression, about the size of a threepenny piece, was found on the extremity of the ileum, and round this was a considerable deposit of lymph. The intestines were replaced in the abdominal cavity and the wound closed. During the night the bowels acted without pain, and with only a trace of blood in the fæces. The patient made an uninterrupted recovery, and went home cured in three weeks. Mr. Bush had seen the patient twice since, at an interval of six months. She remains quite well, and has had no further attack. The eight fatal cases were males. As the mortality in cases of intussusception is so high, abdominal section should be performed immediately after the failure of one rectal injection which had been given slowly and carefully.

Mr. W. J. Tivy spoke of the treatment of intussusception occurring in adults beyond middle age, in whom the acute symptoms often subside quickly, and in whom it was possible, as he had found from experience in two cases, to give a prolonged trial to opium and rectal inflation with a good hope of success.

Mr. Dacre related three cases of intussusception which had come under his observation. The first was that of a child of four years, in which there was no difficulty in making a correct diagnosis. The patient was seen early in the attack, and all the symptoms were at once relieved by inflating the lower bowel with air through an ordinary bellows. The second case was that of a youth of eighteen, who died a few hours after admission to the hospital with all the symptoms of acute peritonitis. The *post mortem* examination revealed a general and severe peritonitis, and in addition an extensive intussusception of the ileum into the ascending colon. This had evidently been started by the weight of a polypus dragging one portion of intestine into another. There were fifteen to twenty polypi in the ileum and colon, of various sizes. The third case, a child of three, was first seen two days after symptoms of acute intestinal obstruction had shown themselves. The physical signs all pointed to intussusception of the bowel as the cause of the obstruction. After insufflation and injection of warm water had been first tried without effect, the abdomen was opened, and an artificial anus made in the distended ileum, as the intussusception could not be reduced, and the patient's condition did not permit of any prolonged operative interference. The child died sixteen hours after the operation from collapse, and at the autopsy a short intussusception of ileum into the colon was discovered. The involved portion was extremely congested, adherent on all sides, and quite incapable of reduction. In cases of intussusception an attempt should always be made at the earliest possible moment to reduce the displacement by means of insufflation of air, or by the steady injection of warm water or warm gruel. Failing this, no time should be lost in performing abdominal section and exposing the involved portion of bowel. If the local condition admitted of safe and easy reduction, this should be attempted: but if not, an artificial anus should be made at the lowest point of the distended gut, in order to temporarily relieve the urgent symptoms. If the patient's condition permitted a more lengthy interference, the affected portion of gut should be resected.

Dr. C. ELLIOTT narrated the case of a boy under his care who, twenty-four hours after a sudden attack of violent abdominal pain and sickness, passed blood *per anum*, and was admitted into the Children's Hospital. Under chloroform a "sausage-like" tumour, from two to three inches long, could be felt in the left iliac region, but not by the rectum. Injection of air with an ordinary kitchen bellows was tried, with at the same time upward kneading of the abdomen. As no improvement resulted in ten minutes, warm water was injected; but as not more than a pint could be thrown up, the injection of air was resumed and continued for fifteen minutes. As no good result was obtained, a consultation was held three hours after, when it was decided to open the abdomen. Before this was done, Mr. Norton suggested the trial of injection of cold water. This was attended with complete success. The tumour entirely disappeared. The patient was put to bed and given an opiate. Next morning the bowels acted naturally. In about ten days the boy was discharged perfectly well, and has remained so since.

Dr. JAMES SWAIN said that in the treatment of intussusception, operation should be resorted to as early as possible after inflation and other milder measures, to which Mr. Prichard had referred, had been tried without success. It is just as important to operate early in intussusception as in other cases of internal strangulation, after the failure of simple methods. The reason of this is at once apparent if we for a moment consider the pathological conditions, which may lead, even within twenty-four hours, to a considerable outpouring of inflammatory lymph between the peritoneal surfaces of the intussusception, and quickly prevent the possibility of disinvagination. It is then the duty of the surgeon to urge an early interference where operation is necessary; but in actual practice we must meet with cases where, from the reluctance of the patient to submit to operation, or from difficulties which precluded an early diagnosis, we find on operation that it is impossible to withdraw the invaginated bowel from the intussuscipiens. Under these circumstances the best operation is that which was originally proposed by Barker,[1] which in simplicity and perfection is much to be preferred to the formation of an artificial anus or the ordinary method of resection of the gut. By means of a diagram Dr. Swain described this operation, and displayed its various steps on portions of intestine in which intussusception had been produced on the cadaver. By utilising the adhesions which prevent the reduction of the intussusception, the continuity of the canal is secured. The operation cannot be done in less than half an hour, and therefore takes longer than the formation of an artificial anus, which is, of course, the only thing to be done where the patient is too much exhausted to allow the necessary time for the more satisfactory operation.

Dr. AUST LAWRENCE narrated the case of W.H., aged six years, who after an attack of diarrhœa lasting four hours, had sudden pain in the abdomen, which persisted for six hours, with blood and mucus passing *per anum*. A finger in the rectum detected an ovoid tumour, which was invaginated bowel. This was reduced under opium, rectal injections of warm water, and massage of the abdomen. These three methods of treatment are of the greatest importance in such cases.

Dr. J. G. SWAYNE showed two water-colour drawings (which he had made many years ago) from specimens of intestinal invagination obtained during *post-mortem* examinations. One of these was from a patient in St. Peter's Hospital, who was under the care of the late

[1] *Lancet*, 1892, vol. i., p. 79.

Dr. William Budd. In this case the invaginated portion consisted of the lower portion of the ileum, the cæcum, and a portion of the ascending colon. This is the most common form of intussusception, and occurs in fifty per cent. of these cases. The invaginated portion is of a deep port-wine colour, as if gangrene were impending. The other drawing represents intussusception of the rectum, a somewhat rare disease; for though the rectum constantly forms the receiving layer of an extensive intussusception, it is very seldom primarily affected. The invaginated portion consisted of the first part of the rectum, and had passed down into the hollow of the sacrum, nearly to the anus. Though apparently within reach of the finger, the nature of the disease had not been recognised during life. The invaginated portion was exceedingly vascular, of a granular appearance, and bright scarlet colour, appearing under the microscope to consist of thickened and hypertrophied mucous membrane. This case was that of an old man, a private patient of the late Dr. Symonds, who had for some time been the subject of chronic diarrhœa, which no medicines or injections would check. Had the precise nature of the disease been made out, surgery might possibly have effected a cure.

Mr. H. F. Mole reported some experiments made in the *post mortem* room in reference to the distension of the intestines in the treatment of intussusception. In each of the first five cases the abdomen was opened by a median incision from xiphoid to pubes, the rectum was then freed as low down as possible, and a long indiarubber tube connected with a glass funnel passed in at the anus and tied firmly into the lower bowel. Water in measured quantity was then poured into the funnel, and the height of this above the table on which the body lay carefully estimated. The intestines were closely watched while the distension took place. (1) **Male child, æt. 11.** Hydrostatic pressure, three feet. After the first pint, the large intestine being fairly distended, the water passed the ileo-cæcal valve with ease, distending the small intestine. This experiment was not carried further. (2) **Female child, æt. 5.** The bowel in this case was first distended with air, by means of a Higginson's syringe, the distension in consequence being of an intermittent character; but eventually the large bowel was evenly distended, and the air passed the ileo-cæcal valve with ease, and commenced to distend the small intestine. (3) **Same subject as in the previous experiment.** Hydrostatic pressure, two feet. After three-quarters of a pint of water the ileo-cæcal valve was passed easily; and the pressure being raised to four feet, after the passage of five pints the transverse colon ruptured, the water issuing from a small hole. (4) **Female subject, æt. 54.** Hydrostatic pressure, three feet. After three pints, water passed through the ileo-cæcal valve, but not at all readily. With a pressure varying from three to four and a half feet, and after seven pints had passed, the transverse colon ruptured near the splenic flexure. The peritoneum first split over a considerable length of the gut, and the hole in the gut was quite a small one, the water issuing in a fine jet. The small intestines were only slightly distended. (5) **An infant, æt. 11 months.** Hydrostatic pressure commenced at eighteen inches, and subsequently raised to three feet; after one and a half pints the valve was passed. The funnel was eventually raised to six feet, but four and a half pints having passed, and the intestines being greatly distended, no more would enter. As the abdomen was opened the results cannot well be compared with what would take place in the living subject, as the support of the parietes would obviously make a great difference. There seems no doubt, however, that both water and air can be made to pass through the ileo-cæcal valve with comparative

6

ease, and this when the large intestine is but slightly distended; and that hydrostatic pressure, though more difficult to work, especially in the living subject, is better than injecting, as it distends the gut more uniformly and evenly, and consequently with less chance of injuring any weak point in the intestinal wall, as no sudden shock is thrown on the gut. When distension was carried to excess, the water, rather than pass through the whole length of the small intestines, burst the large gut, owing probably to its fixed position. No water in any case entered the stomach. In the next three cases, the abdomen being unopened, the intestines were distended with Lund's inflator. It was found that a shorter tube would be more convenient, as the ordinary one had a tendency to hitch on the promontory of the sacrum and kink. It was also found in two of the cases that great force was required after the first few pints had been injected, to prevent the water regurgitating; but this perhaps was due to the marked degree of *rigor mortis* present in each case, and consequently the unyielding condition of the abdominal parietes. (6) **Female subject, æt. 62.** Only a few pints could be injected. The abdomen was then opened, and the water seen in the small intestines. On continuing the injection, about eleven pints were passed, and the large gut then ruptured at the splenic flexure, the peritoneum first splitting longitudinally over a foot of the gut. The water first began to leak through, and then a small jet issued, and finally, on still increasing the pressure, a large rent took place, with forcible expulsion of the contents of the bowel. The small intestines were only partially distended. (7) **Adult male.** About six or seven pints were injected, it being impossible to force in more. The abdomen was then opened, and the large intestine found distended, the water having passed, apparently quite freely, through the valve into the small intestine. (8) **Female subject, æt. 34.** In this case the abdominal parietes were comparatively flaccid, *post-mortem* rigidity having almost passed off. After fifteen pints had been injected, the contents of the stomach flowed from the mouth, presumably from being compressed against the diaphragm by the distended intestines, especially the transverse colon, as on resuming the injection no more fluid came from the mouth. Finally, and after considerable force had been used, thirty-two pints were injected, the abdomen being greatly distended; still none came from the mouth. The abdomen was now opened, and it was found that the transverse colon near the splenic flexure had ruptured, and also that there was a tear in the parietal peritoneum to the right of the umbilicus. There was water in the peritoneal cavity, and it was also widely diffused throughout the cellular tissue of the abdominal walls. The peritoneal coat had split longitudinally over a large extent of the large gut, including the whole transverse colon, and in a few places in the small gut. No water had passed into the stomach; indeed, the small intestines might have been much more distended. The only results of these experiments appear to be to show that no damage can be done to healthy intestines by injecting fluid with moderate amount of force; that the ileo-cæcal valve is easily passed in all cases; that in no case could water be made to pass the whole alimentary tract, or even reach the stomach (but the bodies experimented with were all horizontally placed, none inverted); and lastly, that the part most likely to be damaged by distension was in every case the transverse colon, either at or close to the splenic flexure, and before great distension of the small intestines had taken place.

Dr. F. H. Edgeworth drew attention to a specimen which had been brought for inspection from the Museum of the Royal Infirmary.

The patient from whose body it had been removed *post mortem*, had been admitted under Mr. Greig Smith, with the ordinary symptoms and physical signs of intussusception. The "sausage-shaped" tumour disappeared under rectal injection of milk, and the patient appeared to be on the high road to recovery, when he developed some obscure symptoms resembling tetanus, and died. At the autopsy an irreducible partial intussusception of the vermiform appendix into the cæcum was found. Apparently then, the apex of a considerable intussusceptum into the large intestine was formed by this intussuscepted appendix instead of by the usual ileo-cæcal valve. The specimen was believed to be unique. In the treatment of cases in which diagnosis was uncertain, Wilks has laid down the valuable rule, that no patient who was so ill from intestinal pain as to consult a doctor, should be treated by a purgative, unless the disease was, from the physical signs, obviously one of colic.

Mr. Greig Smith congratulated the Society on the general excellence of the discussion, and called special attention to what appeared to him to be the most noteworthy practical points in the remarks of the various speakers. In the treatment of an actual case, opium, as Dr. Lawrence had urged, would probably be employed; it was the only form of intestinal obstruction in which the use of opium was commendable. Then the weight of opinion was towards the employment, as a first step, of distension of the colon by injection of fluid by the rectum. Mr. Mole's experiments in the dead-house were here of conspicuous value, as showing the tensile strain of the colon to hydrostatic pressure, and the degree of pressure necessary to force fluid through the ileo-cæcal valve. It was clear that an elevation of the injecting reservoir of more than three feet was not safe. Hydrostatic pressure from an elevated reservoir should be employed, rather than insufflation by bellows or injection by hand-pressure. Prolonged distension under low pressure was more successful than rapid distension under high pressure. Mr. Bush was to be congratulated on being the only one of their number who had succeeded in saving a patient by operation. In that large proportion of cases where reduction was impossible, Barker's ingenious method of operation should be adopted. Mr. Greig Smith further called special attention to the admirable display of specimens exhibited, and thanked the curators of the Museums of the Royal Infirmary, General Hospital, and Children's Hospital for their loans. Every variety of intussusception was shown, some varieties being unique in their rarity or excellence. One of the Medical School specimens which he had cut vertically showed the anatomy of the condition very clearly. The beauty of Dr. Swayne's drawings was commented on, and a hope expressed that they might find their final resting-place in the School Museum. By the aid of a drawing, and by a working intestinal model on which intussusception was produced, Mr. Greig Smith dwelt upon certain practical points in the production and reduction of the conditions. The crossing of the mesenteric folds, limiting the descent of the intussusceptum and the ascent of the intussuscipiens; the plication of the ascending and receiving gut, which could be undone only by pulling downwards the receiving bowel; the small size of the actual intussusceptum and its influence in continuing the condition were demonstrated and discussed. Finally, some suggestions as to detail in Barker's operation were made.

### British Medical Association.

### Bath and Bristol Branch.

*January* 31*st*, 1894.

Dr. R. Shingleton Smith, President, in the Chair.

The evening was devoted to a discussion on Appendicitis, introduced by a paper by Dr. James Swain. (See page 9.)

Mr. W. H. Harsant said: The simplest way of classifying cases of appendicitis is to describe them as non-perforative or perforative, and to sub-divide the latter into circumscribed and diffuse. Suppuration is nearly always the result of perforation; recurrence simply means two or more separate attacks of the same illness, while relapsing appendicitis is only a variety of the non-perforative kind, with intervals of partial recovery between the acute attacks. The treatment of appendicitis resolves itself mainly into the question of operation. Shall we operate or not? And if so—when, and how? We might begin by laying down the rule, that when the appendix is known to be peforated we should operate without delay; but the converse is not true, for we should sometimes operate when there is known to be no perforation. All would agree that cases of diffuse perforative appendicitis should be operated on at the earliest possible moment, for these cases rarely live longer than a week unless operation be performed, and they are nearly always fatal even then. A few years ago I operated in a case of this kind. I was summoned in the night to go to Staple Hill to see a patient in the practice of Dr. Murray. Dr. Beddoe had seen the case that evening, and recommended immediate operation. I found a man who had been ill four days only, with symptoms of acute intestinal obstruction; indeed, the symptoms in this form of the disease always do resemble those of intestinal obstruction. His belly was much distended, and there was evidence of fluid in the peritoneal cavity. I opened the abdomen and let out a quantity of fæcal pus. I washed it out, and inserted a drainage tube; but the patient died the next day, and there was found a perforation of the vermiform appendix, with a diffuse purulent peritonitis. In cases of circumscribed suppuration the result of perforation we ought, it seems to me, to operate as soon as the diagnosis is clear. If pus is in the peritoneal cavity, we never know how soon it may become diffused; and the old idea of waiting until it comes to the surface or points is a very bad one, for the sooner we operate the better for the patient. It is in this class of cases that we are sometimes in doubt whether merely to operate and drain the abscess, or to search out the vermiform appendix and remove it. I have operated on two cases of this kind at the Infirmary during the last few years; and in one of them I was persuaded by a colleague to search for the appendix and remove it, while in the other I simply drained the abscess cavity, and both patients made a good recovery. In any future cases the rule I shall follow is, that if the appendix can be found without tearing down any dense adhesions, or without much disturbance of parts, I shall remove it, but not otherwise. My first case will illustrate the danger of pushing a grooved needle into the abdomen. A girl, aged 20, was sent to the Infirmary, on November 27, 1892, by Mr. Ewens, who had been called in by a medical man to see the case, and who at once recognised it as a case of perforative appendicitis. A swelling existed over the region of the cæcum, which had been punctured with a grooved needle before Mr. Ewens saw the case.

Nothing had come through the needle except gas and a little fæcal matter; but the effect of the puncture was to set up a septic cellulitis in the abdominal wall and the lumbar region. I opened and drained the peritoneal abscess, in which I found a lump of fæcal matter which had come through a perforated appendix. I did not remove the latter, as it was firmly bound down by adhesions; but I made numerous incisions into the cellular tissue of the loin, and the patient recovered, with nothing worse than a slight ventral hernia at the seat of the incision. It is in cases of the non-perforative variety, or where perforation is doubtful, that we are in the greatest difficulty whether to operate or not. Of course much depends upon the gravity of the attack. If the symptoms show signs of great severity, operation should be earlier performed than when the contrary is the case; but in any case operation should never be delayed beyond the second week, unless symptoms of improvement are manifested. Lastly, relapsing cases and cases of repeated recurrence may demand removal of the appendix in the intervals of the attacks; but even here we need not hastily assume that operation is absolutely necessary. I have had the opportunity of watching two patients in private practice, one of whom has had four attacks and the other three, and both have now been free from trouble for several years, and have apparently completely recovered, without operation. These, however, were not relapsing, but merely recurrent attacks, which are not nearly so grave as the severe form of malady to which Treves has called attention, and has given the name of relapsing. Here the only question is when the operation can be best performed, whether during the intervals of the acute crises, or at the commencement of one of the latter. Fortunately these cases are not often met with, and each must be dealt with on its own merits.

Mr. C. A. Morton said: I have been looking over my records of nine cases of perforation of the appendix. I should like first to make some remarks on the mechanism of perforation in these cases. Talamon believes that the concretion blocks the tube and impairs the nutrition of its walls beyond by pressure, and allows mucus to collect in the distal part, which becomes a cultivation fluid for the micro-organism soon destined to penetrate the devitalised wall and set up appendicitis. If this were so, I think we ought to find the concretion still impacted in the tube after perforation beyond it, and we ought to find the distal portion dilated from the distension, as Treves has pointed out so commonly happens when in relapsing cases the appendix is simply obstructed without the presence of a concretion. Neither condition, in my experience, do we find in perforating suppurative appendicitis. I will refer to my *post mortem* records of four cases in which such was certainly not the condition. **Case 1.** Boy, æt. 8. Perforated ulcer, with sloughy base *where concretion lay*, at tip of appendix, allowing pus in the tube to escape. There was certainly liquid fæcal matter in the peritoneum, which must have passed the concretion and escaped at the perforation, as no other perforation could be discovered. This was a case, not of localised peritonitic abscess, but of diffuse purulent peritonitis. **Case 2.** Girl, æt. 7. The tip of the appendix was separated from proximal part by ulceration, but was still attached to the cæcum by adhesions. It was not gangrenous. A few drops of liquid fæces escaped from the proximal end. Close by, in the peritoneal cavity, a putty-like concretion was found. There was no concretion remaining in the appendix. It seems to me most probable that where the concretion had lodged a circular ulceration had been set up, leading to complete severance of the tip, and liberation of the concretion. This, like the last case, was one of general peritonitis. **Case 3.** Male, æt. 18. Appendix was greatly thickened and club-shaped, but not much distended. There were two perforations opposite one another, from both of which sloughs had not wholly separated. Appendix between cæcum and clubbed end normal. It seems highly probable that the concretion (which was not found) lay between the ulcers and escaped through one. A few very minute concretions still lay in the appendix. **Case 4.** Male, æt. 16. The end of the appendix had sloughed completely off and disappeared. The remaining inch of the appendix was quite patent, and mucus exuded from it. I will now refer to some of the clinical features in these cases, and first with regard to my own case—Case 4 of the pathological reports. There was a large swelling, reaching from the anterior superior spine to the umbilicus; and yet, after thirteen days' illness, no adhesion had formed to the abdominal wall. I made my incision in the usual place, just inside the anterior superior spine, but had to open the peritoneal cavity, and a part of the cæcum of normal appearance presented. From this wound, however, I could feel a hard irregular mass just within, with a soft spot in the centre; and I made another incision in the semilunar line immediately over it, and found an encysted collection of pus which had already ruptured into the peritoneum. The second case which I should like to refer to was admitted into St. Bartholomew's Hospital when I was house-physician there, and was one of general diffuse purulent peritonitis from the first, in a young man. But there was no tenderness of the abdomen whatever, and the vomiting was fæcal, conditions which led to a diagnosis of mechanical intestinal obstruction rather than peritonitis. Perhaps the most interesting of all these cases is one in which, *post mortem*, I found the perforated appendix hanging over into the cavity of the true pelvis, and an abscess formed there shut in by adherent coils above. There was no iliac swelling, but the pelvic one could be felt *per rectum* during life. The abscess had burst into the general cavity of the peritoneum, and general peritonitis had set in. I did not see the case during life, but made the *post-mortem* examination. It is Case 3 in the pathological records. Sometimes a perityphlitic abscess may be resonant, from gas generated by decomposition of the pus. I had under observation a case of this kind in the Children's Hospital. There was the typical swelling and symptoms. It was punctured by an exploring needle, and nothing came. It was very cautiously incised, and stinking pus was evacuated; and the boy recovered. I have quite lately opened a large pelvic abscess which was resonant from contained gas, which rushed out on opening it; and some time ago I saw, in consultation, a pelvic abscess pointing in the groin, which, because it was resonant, was supposed to be a hernia.

Dr. H. Waldo said: It appears to me that there is in some cases of appendicitis a cause which Dr. Swain has not touched upon. It is a gouty inflammation of this part of the intestine; a cause which, I think, is generally ignored by surgeons. Sir Alfred Garrod gives notes of the case of a very gouty man, aged 50, in whom, after exposure to cold, gout retroceded to the intestines, producing intense inflammation of the last eighteen inches of the ileum, as found after death. Another authority on this subject (Dr. Haig) considers that a large number of cases clinically indistinguishable from typhlitis, depend upon a gouty inflammation of the fibrous tissues of the intestinal walls, and he reports more than one case successfully treated by salicylate of sodium. I once treated upon this plan an acutely painful and tender enlargement of the cæcum in a gentleman over seventy years of age. The pain was very much relieved by salicylates, and the patient soon recovered. A powerful argument against very many cases of typhlitis depending upon uric acid, is the age of the patient; typhlitis mostly occurring before the gouty age. But, I think, with a reasonable suspicion of gout, I should give salicylates a trial, and, if possible, avoid opium and belladonna, as they so mask the symptoms, before handing the case over to the operating surgeon.

Mr. Pagan Lowe considered that the clinical aspect of appendicitis, and the *post-mortem* appearances, were insuperable objections to the theory that foreign bodies produce the disease. The lymphoid tissue which surrounds the vermiform appendix is analogous in structure to the lymphoid tissues of the tonsils, and, like it, is remarkable for the variety of its inflammations. As in tonsillitis only a few cases go on to suppuration, so also in appendicitis abscess is the exception and not the rule. Appendicitis has been described as abdominal tonsillitis, and this description may be the correct one. Should it prove to be so, an explanation is at once afforded of the recurrent and relapsing forms of the disease. In cases not requiring operation, treatment by absolute rest, a diet consisting of nothing but hot water flavoured with brandy and Valentine's beef-juice, and the administration of salicylate of soda (in fifteen-grain doses three times a day) has given excellent results. Opium should only be given when the pain is severe, and enemata and aperients not at all.

Dr. Charles Steele said: In considering this subject two great difficulties arise—the first that of accurate diagnosis, and the second that of performing a reliable operation. When cases follow the usual course of giving warning a short time previously, by irritation in the right iliac region, all other causes can be cleared off as not producing it, and we are able to define, in all probability, the reason of obstruction as lying in the vermiform appendix. But where cases occur suddenly without previous warning, and closely simulate those conditions which depend upon obstruction of the colon by an accumulation of feculent matter, the diagnosis is often impossible until operative measures can be of little avail. Such was the following case: A lady, aged 49, was taken suddenly ill on the 26th December, with severe pain in the abdomen. I saw her very promptly, and found her suffering from subacute peritonitis, with a very loaded tongue. There had been no rigors, and gentle manipulation did not enable me to pick out any one spot of particular tenderness. Associated with this was sudden and extreme sickness. There was no accumulation in the rectum, but at the highest point that could be reached the passage was contracted by pressure external to itself, as the mucous membrane was in a perfectly healthy state, and a large soft formation could be felt around it. I explained without hesitation that an operation would be necessary; and Mr. Harsant saw her within an hour with me, and we arranged an operation in the early afternoon. This, with Mr. Bush's assistance, as well as Mr. Harsant's, I performed, opening the walls in the linea alba, and on perforating the peritoneum a thin purulent offensive fluid escaped. I introduced two fingers, breaking down a few adhesions to the bowels, and opened up a large accumulation of the same fluid, together with lymph, in the left iliac region, which was making an attempt to become an abscess, and was the cause of the constriction of the rectum. These fluids having been thoroughly washed away by syphonage, I drew out a portion of the small intestine, stitched it carefully to the wound, put in a drainage tube, and on opening the intestine a fair amount of feculent matter was evacuated. The patient, on recovering consciousness, was much relieved, and had a further evacuation three hours afterwards; but collapse supervened, and she died the next morning at 2.30. Mr. Bush and I made a *post mortem* examination, and found the wound in a perfectly satisfactory state; that nothing had run back into the abdominal cavity from it, and we could discover no cause of obstruction until I drew out the vermiform appendix, which had a small, hard feculent mass in it, and a small opening near the extremity with well rounded edges, through which a tortoise-shell hairpin could be introduced. This was the secret of the whole illness. Now the correct operation in this instance, as there had been no pain or other indication in the right side, was performed; but, as often happens, the *post mortem* examination made one feel very humiliated—for whereas everything seemed to have been done that could be done, yet the real trouble was entirely unmastered. Of this I am determined, that when I have another similar case where operative interference becomes requisite and yet perforation of the vermiform appendix does not appear to be the probable cause of the necessity for operation, I shall not be satisfied until I have examined that organ—and removed it, if the fault exists in it.

Dr. Michell Clarke said that as the previous speakers had discussed appendicitis from the surgical aspect, he would make a few remarks from the physician's point of view. The first point that occurred to him was, that only a very small proportion of cases of perityphlitis would require surgical procedures if the diagnosis was always made at once, and appropriate treatment put in force. Hence the great importance of making an early diagnosis. This is often difficult. The pains of commencing perityphlitis may be general, simulating general peritonitis. Thus, in a case under his care, a girl of eighteen was seized with violent pains and diarrhœa within twenty-four hours after swallowing a sharp and large plum-stone. The pains were felt over the whole abdomen, which was distended and tender. Twenty-four hours later pain was localized, and confined to the right iliac fossa. Subsequently a large tumour formed there. The patient recovered after an illness of about four weeks, and as the swelling subsided a small hard elongated body could be felt in the position of the cæcum, which was either the plum-stone or the appendix. This girl has remained quite well during the two and a half years that have elapsed since the illness. Another point was the occurrence in his experience almost invariably, not of constipation, but of the passage of two or three or more loose stools at the onset of the illness. Then in some cases it may be difficult at first to distinguish appendicitis from typhoid fever. Thus, a man under his care suffered from perityphilitis with portal pyæmia, and secondary abscesses in the liver. This complication made the case resemble enteric fever at first, and an accurate diagnosis could not be made until nearly two weeks after the onset. Dr. James Swain has mentioned the views held as to the part played by the bacterium coli commune in appendicitis; and I may incidentally mention that Mr. Stoddart kindly made cultivations for me from the pus in this case, but found only the common pyogenic organisms. In another case of portal pyæmia, secondary to dysenteric ulceration of the colon, the bacterium coli commune was present. Further, cases occur in women in which inflammatory exudation affects both the pelvis and the right iliac fossa, and in which it is difficult to say whether the peritonitis started from the pelvis and spread upwards, or *vice versâ*. That the cæcal form of perityphlitis from fæcal accumulation has been altogether disproved he did not believe, although the nature of the cases does not so readily admit of proof *post mortem* as in the case of appendicitis. A case of perityphilitis in an old lady of 88, who suffered from habitual constipation, appeared to be certainly of this nature: it ended in recovery. As to the general treatment of ordinary cases of appendicitis, he thought the administration of aperients and enemata was inadvisable. It had been shown over and over again that patients do not suffer any harm from not having an evacuation of the bowels for two or three weeks, or even longer; whilst there was certainly risk, which could not be guarded against, because its exact nature could not be foreseen in each individual case, in administering purgatives and enemata. There was no reason to depart from the established treatment by absolute rest and opium. With regard to the indications for operation. In a case seen from the first, the pulse-rate and the temperature should fall and remain normal after the first two or three days if the patient was doing well: but if after the temperature had been normal for some little time it began to rise again and remain high, and if at the same time the pulse became worse in character, then it was time to operate. If, on the other hand, the patient had been ill for some time before coming under observation, the question of operation must be decided on general principles. If in either case there is a doubt as to the presence or not of pus, but the patient is not doing well, is losing ground under ordinary medical treatment, then it is better in such a case of doubt to operate without further delay. It is well to remember that even desperate cases may recover by means of operation. A boy in whom perforation into the general abdominal cavity had presumably taken place two days before he was seen, whose abdomen contained free pus and gas, and who apparently could not live twenty-four hours, recovered perfectly after operation. The abdomen was simply opened and washed out by Mr. Paul Bush, who a few days later, at a second operation, removed the appendix. Lastly, the great success of operative procedures in cases of appendicitis during recent years, should not make us forget that the large majority of cases of appendicitis do not require operation, or make us less careful and painstaking in carrying out the ordinary medical treatment by rest and full doses of opium.

Mr. Nelson C. Dobson said: I wish to confine my remarks for the most part to some points in the treatment of appendicitis: the difference of opinion which has already been expressed seems to me to arise in a great measure from the fact that the disorder under consideration is one of variable intensity, and the observer naturally draws his inferences from the class of cases he is most frequently meeting with. Now, without attempting any perfect or precise classification, I think we may, for practical purposes, divide appendicitis into four classes: (1) Those cases with the ordinary symptoms and without definite swelling in the iliac region, and which with judicious medical treatment recover and remain permanently well. (2) Those in which the symptoms are more marked, and have added to them a distinct swelling, and which go on to suppuration: these cases require to be opened at the most convenient spot as soon as pus is found. Generally simple evacuation and drainage is all that is necessary; the appendix should only be removed in such cases when this is easily within reach. (3) Those that are intermediate between the first two, and neither suppurate nor get permanently well. It is in "relapsing" cases that removal of the appendix during a quiescent period is practised with more or less frequency and success; but looking back on my past experience, I think the occasions in which it is necessary must be very rare. I have found that by rest and patience, thickened and inflamed appendices thoroughly recover. I have also found that adherent, distorted, and displaced appendices in several cases gave rise to no discomfort during life. The last class is the perforating or fulminating variety. Here the only chance of success lies in operation. When general peritonitis is present the abdomen should be opened and drained, without attempting to remove the appendix. If we could diagnose the exact moment of perforation, we should cut down on the appendix and remove it; but I know of no precise means of diagnosing its occurrence, and I am not prepared at present to advocate the rule in strangulated hernia—" When in doubt, operate."

Mr. Greig Smith dwelt upon the difficulty of working out a classification of appendicitis which would be at once scientific and practical. The natural groupings of the disease as found in the *post mortem* room or on the operating table were exceedingly varied, and did not lend themselves to definite classification. Practically he had found in his own experience of some fourteen operations exceptions to the classification he had adopted. Thus he had found perforation in a case of relapsing appendicitis; and an enormous abscess was found around a gangrenous appendix which had not perforated. After remarking on the influence which the foreign body played, and giving an explanation of various features in the disease which had struck him, he thought that in their practical work they would find it convenient to recognise three leading varieties; viz.: (1) The fulminating or perforating, with fæcal extravasation, almost uniformly fatal; (2) the suppurating, such suppuration being nearly always localised, and demanding immediate operation; (3) the simple, usually plastic, but often merely catarrhal, and occasionally relapsing, and demanding operative relief according to the urgency of the objective symptoms.

Mr. Paul Bush said that we owed a great deal to American surgeons; but he wished to call attention to the fact, that in the records of the Bristol Royal Infirmary of sixty years ago the late Mr. Fred. Granger, of this city, described very accurately the position of what is now known by the name of "McBurney's spot." This "spot," if it must have a special name, should be called the "Bristol spot" or "Granger's spot."

Mr. W. M. Barclay laid stress on the importance of early and persistent medical treatment, especially the administration of opium. The chief indication for operative interference (excluding fulminating and relapsing appendicitis) is the advent of suppuration; and the determination of this cannot be obscured by the drug, whereas it may have a directly curative effect by relaxing the spasm that may produce kinking of the appendix and by abolishing peristalsis. In the relapsing form, operation is advisable if the case has been watched sufficiently long and is not improving, or shows signs of increasing in severity or frequency of recurrence.

Mr. Pickering said that to state that an operation was necessary in the suppurative form of appendicitis did not give any practical guide as to when an operation should be performed, because it was often very difficult to know when suppuration existed, and minor cases of the suppurative form of appendicitis would recover without operation. He considered that an operation was necessary—(1) in cases where fluctuation could be felt in the swelling; (2) in cases where, notwithstanding appropriate medical treatment, the temperature kept persistently high, or where the swelling increased in size either with or without a high temperature; (3) in cases where the exploring syringe had been used, and pus tapped.

Dr. W. H. C. Newnham deprecated operative interference in every case of appendicitis, and said that although he had seen an enormous number of such cases during eleven years' experience as a hospital resident, he had never known a fatal case, nor one in which operation was necessary.

Dr. G. Parker said that the cause of appendicitis appeared to be entirely unknown. Some occasional poison in the fæces might be the agent. Certainly neither concretions, strictures, colitis, nor the mechanico-bacillary theory of Talamon would account for it. Ribbert and Fitz had shown that concretions were actually found oftener in healthy appendices than in diseased ones, nor were they always at the affected spot, nor was it clear how they could possibly cause the inflammation. Strictures, partial or complete, had been found in 25 per cent. of healthy persons; and, in fact, the orifice of the appendix gradually becomes smaller with advancing years. Though Hodenpyl recorded the presence of the bacillus coli communis in 33 out of 35 cases of perforative appendicitis, that was simply because it alone survived in the cultures. Borlacci showed that there were plenty of others, and that the bacillus coli alone would not cause peritonitis.

Dr. Shingleton Smith remarked that as regards the treatment of appendicitis, the two things needful were rest and opium to relieve pain. There was no objection to the administration of opium in cases where all question as regards obstruction of the bowel could be excluded, and in cases where the pain is severe it must be absolutely impossible to withhold opium or morphia in sufficient quantity to give relief to the pain; at the same time it tends to keep the bowel in absolute rest, so as to favour adhesion and so prevent general peritonitis when perforation may occur. The use of saline aperients and enemata should not be encouraged, as they were in opposition to the principle of keeping the bowel at absolute rest. Peptonised foods should also be given on the same principle; viz., that of encouragement to alimentary inaction.

